# Supplementary material for: Wien effect in interfacial water dissociation through proton-permeable graphene electrodes
Source: Nat Commun. 2022 Oct 1;13:5776. doi: 10.1038/s41467-022-33451-1 (PMC9526707; doi:10.1038/s41467-022-33451-1)
Supplement: Supplementary file 1 — Supplementary Information [file 41467_2022_33451_MOESM1_ESM.pdf]

Supplementary Information

Wien effect in interfacial water dissociation through proton-permeable graphene electrodes

J. Cai[1,2,3*], E. Griffin[1,2*], V.H. Guarochico-Moreira[1,2,4], D. Barry[2], B. Xin[1,2], M. Yagmurcukardes[5,6], S. Zhang[7], A. K. Geim[1,2,8], F. M. Peeters[5], M. Lozada-Hidalgo[1,2,#]
* These authors contributed equally
[1]National Graphene Institute, The University of Manchester, Manchester M13 9PL, UK
[2]Department of Physics and Astronomy, The University of Manchester, Manchester M13 9PL, UK
[3]College of Advanced Interdisciplinary Studies, National University of Defense Technology, Changsha, Hunan 410073, China
[4]Escuela Superior Politécnica del Litoral, ESPOL, Facultad de Ciencias Naturales y Matemáticas, P.O. Box 09-01-5863, Guayaquil, Ecuador
[5]Departement Fysica, Universiteit Antwerpen, Groenenborgerlaan 171, B-2020 Antwerp, Belgium
[6]Department of Photonics, Izmir Institute of Technology, 35430 Urla, Izmir, Turkey
[7]Key Laboratory for Green Chemical Technology of Ministry of Education, School of Chemical Engineering and Technology, Tianjin University, Tianjin 300072, China
[8]Centre for Advanced 2D Materials, National University of Singapore, 117546 Singapore
[#]Corresponding author. E-mail: marcelo.lozadahidalgo@manchester.ac.uk

This file contains:

- **Supplementary Figures 1-5**

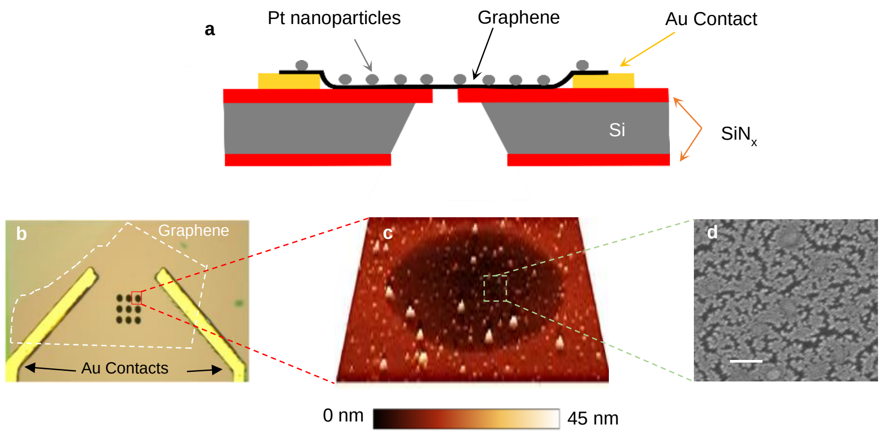

Supplementary Figure 1| Device geometry. a, Schematic of the devices used in this work. **b**, Optical image of one of our devices (top view). Black circles, 2 µm diameter apertures in the SiN_x membrane. White dotted lines mark the area covered by monolayer graphene. Devices typically had two gold electrodes in order to ensure good electrical contact with graphene (usually they were shorted). **c**, Atomic force microscopy image of the area marked with a red square in panel **b**. The Pt nanoparticles coalesce to form discontinuous films that appear as irregular features in the image topography. These features have a typical height of a couple nanometres with some large agglomerations of tens of nanometres. **d**, Scanning electron micrograph of the area marked in panel **c** shows that the films were formed by individual nanoparticles a couple nanometres in size. Scale bar, 20 nm.

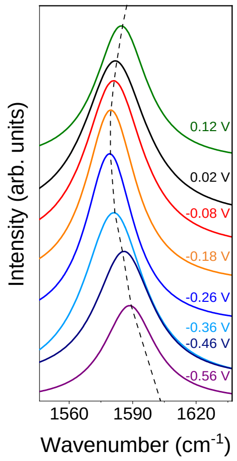

Supplementary Figure 2| Probing of carrier density in graphene electrodes by Raman spectroscopy. Examples of Raman spectra of the *G*-peak for graphene electrodes as a function of potential vs silver-silver/chloride reference electrode. The position of the *G*-peak reaches a minimum around 1576 cm^{-1} and blue shifts as a function of *V*-bias. Spectra are shifted along the *y*-axis for clarity. Dotted curve, guide to the eye.

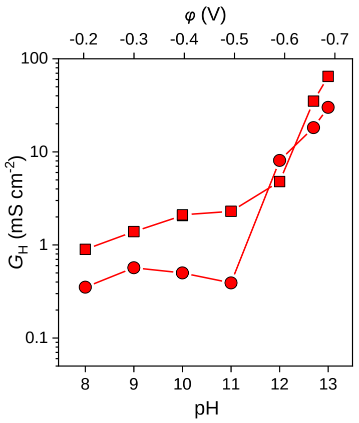

Supplementary Figure 3| Reproducibility of the *I-V* response over the pH range. Two sets of data of proton conductivity from a typical device. Different symbols mark different measurement sets. Both measurement sets run from low to high pH. At the end of each run, the device is flushed repeatedly with deionised water and then left in it for a couple of hours before the next run. Top *x*-axis, zero current potential φ(pH) relation measured against the silver-silver/chloride found for graphene devices.

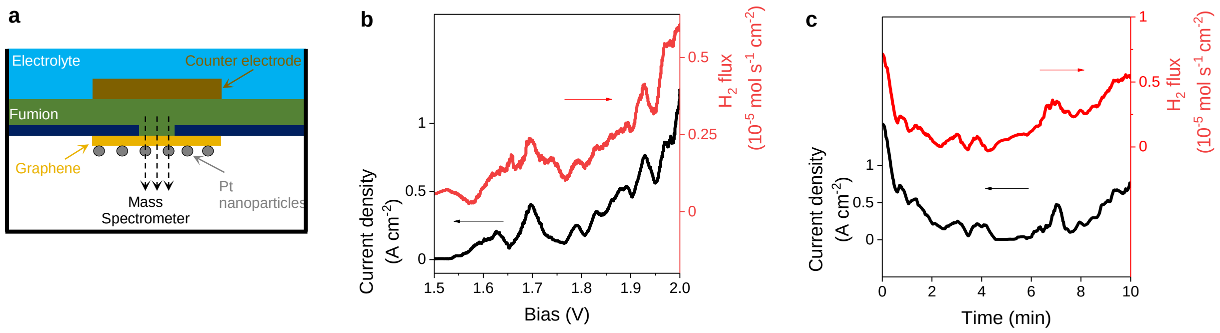

Supplementary Figure 4| Hydrogen Faradaic efficiency experiment. a, Schematic of the experimental setup. **b**, Example of current density and hydrogen flux measurements recorded simultaneously as a function of applied *V*-bias. **c**, Example of current density and hydrogen flux measurements recorded as a function of time. Solution pH 11.

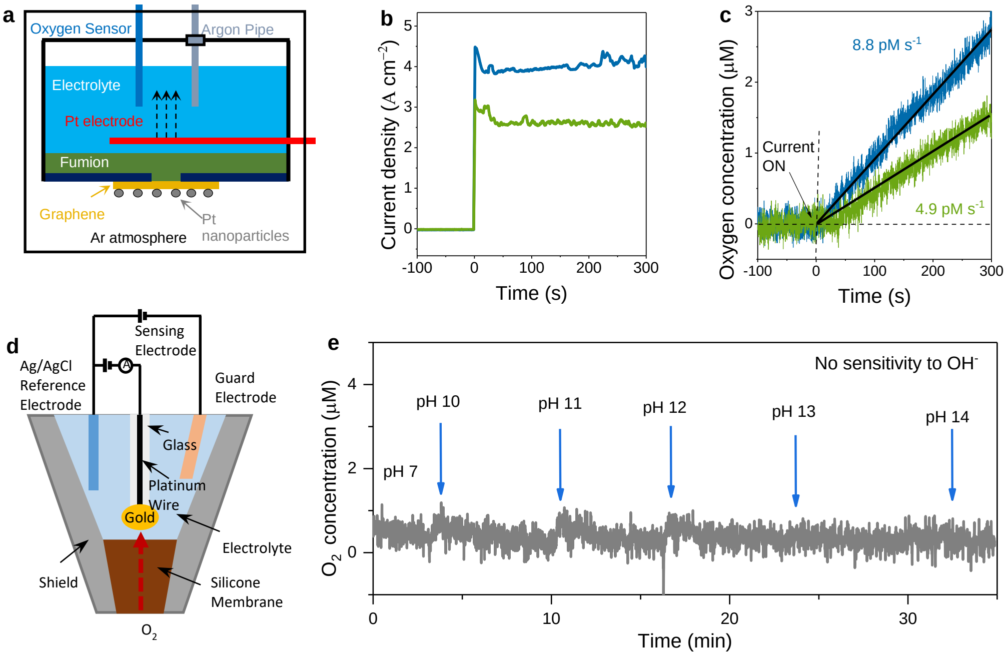

Supplementary Figure 5| Oxygen Faradaic efficiency experiment. **a**, Schematic of the setup. The oxygen evolution reaction takes place at the Pt counter electrode (marked in red). **b**, Examples of current vs time in graphene devices for V = 2.6 V (green) and V = 3.2 V (blue). The current is approximately constant over time. **c**, Oxygen concentration in the electrolyte vs time recorded simultaneously while recording electrical current in panel **b**. The concentration increases linearly with time, from which we deduce the rate of O_2 evolution. Solution pH 11. **d**, Schematic of the oxygen sensor. **e**, Oxygen concentration vs time in the control experiment showing that the sensor does not detect OH^- ions. The arrows mark the time at which a given pH was reached in the tested solution after the introduction of Ar-saturated KOH stock solution.
